# Parents' intentions toward preschool children's myopia preventive behaviors: Combining the health belief model and the theory of planned behavior

**DOI:** 10.3389/fpubh.2022.1036929

**Published:** 2022-11-24

**Authors:** Nan Jiang, Jiayue Chen, He Cao, Yongyi Liu, Yuxin Zhang, Quqin Wang, Ting Wang, Huilan Zhao, Hui Lu, Lei Yang, Jiwei Wang

**Affiliations:** ^1^Key Lab of Health Technology Assessment of Ministry of Health, School of Public Health, Fudan University, Shanghai, China; ^2^Huacao Community Health Service Center, Shanghai, China; ^3^School of Public Health, University of Washington, Seattle, WA, United States

**Keywords:** health belief model, theory of planned behavior, myopia, preschool children, parental intention

## Abstract

**Objective:**

This study aimed to develop an integrated model based on the health belief model (HBM) and the theory of planned behavior (TPB) to explore the influencing factors of parents' intentions toward preschool children's myopia preventive behaviors.

**Methods:**

This cross-sectional study was conducted in Minhang District, Shanghai, China in January 2022. One thousand six hundred and twenty-eight parents of preschool children from seven preschools were recruited in the study. A four-part questionnaire was used to collect data on socio-demographic characteristics, HBM variables, TPB variables and parental intentions. This study used exploratory factor analysis to analyze HBM and TPB items. Hierarchical multiple regression analysis was performed to explore the relationship between independent variables and parents' intentions toward preschool children's myopia preventive behaviors.

**Results:**

The final integrative model showed that perceived severity, perceived barriers, attitudes, subjective norms, and perceived behavioral control were associated with parents' intentions toward preschool children's myopia preventive behaviors. In model 1, Child's age was entered as a control variable and explained 0.6% of the variance (*F* = 7.241, *p* = 0.007). When the HBM variables were entered in model 2, the proportion of variance increased to 25.4% (*F* = 73.290, *P* < 0.001). In model 3, TPB variables were entered and explained 63.2% of the variance (*F* = 246.076, *p* < 0.001).

**Conclusion:**

The integrated model of HBM and TPB constructed in this study significantly improved the degree of explanation of parents' intentions toward preschool children's myopia preventive behaviors. Parents' perceived severity, perceived barriers, attitudes, subjective norms, and perceived behavioral control can be prioritized intervention targets for myopia preventive practices in preschool children.

## Introduction

Myopia is a growing public health concern, especially in Asian countries due to its high prevalence (1). A recent study showed that the prevalence of myopia in 4-year-old, 5-year-old, and 6-year-old children in China was 5.7, 5.8, and 7.1%, respectively (2), higher than the finding from Australia (1.4%) (3) and Kosovo (3.4%) (4). Most importantly, previous studies have shown that the earlier the onset of myopia, the more likely it is to progress into more severe myopia and even lead to irreversible loss of vision (5, 6). For example, the increased risk of myopic complications such as myopic macular degeneration, retinal detachment, cataract, and open angle glaucoma, have become the leading cause of untreatable visual loss in East Asian countries (7, 8).

Although the cure for myopia has not yet been established, the risk of myopia progression is manageable. There is empirical evidence that both genetic and environmental factors contribute to the development of myopia (9, 10). As modifiable environmental risk factors, myopia preventive behaviors are receiving increasing attention. Myopia preventive behaviors were divided into two categories. The first is the risk behaviors for myopia, such as prolonged screen exposure, prolonged reading and writing, and prolonged near work (10). The second category is protective behaviors for myopia, such as plenty time for outdoor activities (11). Restricting individual risk behaviors and promoting protective behaviors will help prevent the development of myopia (1, 12). It has been well-documented that the period from birth to 3–5 years of age is critical for visual development (13, 14). However, because preschool children have poor self-control, they always tend to follow their parents' arrangements (15, 16). Thus, near work, screen and outdoors time among preschool children are significantly influenced by parental attitudes and intentions (16).

Complicated psychosocial factors influence parents' intentions toward preschool children's myopia preventive behaviors, such as self-efficacy, response efficacy, and susceptibility (17). Therefore, a single theoretical model has limitations in explaining behavioral intentions (18). Under this circumstance, many researchers begin to explore the possibilities of combining various theories to construct a more comprehensive theoretical system to explain behavior or behavioral intentions. For example, one study constructed an integrated model of the theory of planned behavior (TPB) and self-determination theory to explain myopia-preventive behaviors (19). In addition, some researchers have proposed the comprehensive application of TPB and health belief model (HBM) interpretation of parents' intentions to vaccinate their children (20).

TPB is one of the most widely used social cognitive models that attempts to predict behavioral intentions and health behaviors (21, 22), which is an extension of the theory of reasoned action (23). Specifically, TPB posits that intention is the immediate predictor of behavior, which is determined by attitude, subjective norm, and perceived behavioral control. This theory states that when individuals perceive a behavior as positive (attitude), know that important people want them to perform the behavior (subjective norms), and perceive that the behavior is under their own volitional control (perceived behavioral control), then the individual will perform the behavior (24). The TPB has been applied to many health behaviors including myopia preventive behavior and parents' intentions to vaccinate their children (19, 25).

The HBM is a theoretical guideline and general conceptual framework for health behaviors (26). HBM focuses on changes in health beliefs, which lead to changes in health behaviors (27). According to the HBM, individuals' involvement in health-related behaviors can be explained by perceived susceptibility, perceived severity, perceived benefits, perceived barriers, self-efficacy, and cues to action. The theory postulates that individuals are more likely to adopt healthy behaviors when they perceived risks and believe these behaviors can promote their health (28). HBM has been widely used in various health behaviors, including myopia preventive behavior intentions (29, 30).

HBM and TPB are two of the most common and accepted theories in the field of health behavior. Both of them are based on the expectancy value theory, but offer different perspectives on behavioral decision-making (28, 31). However, a number of studies have identified that existing single theories may have some shortcomings that potentially reduce their predictive power (19, 32). For example, TPB is considered not to identify the more global cognitive variables related to its constituent variables and the model does not account how general motivation serves as a source of information to guide social cognitive processes (32). One of the limitations of HBM is that it ignores the influence of external pressures such as subjective norms on one's acceptance of a healthy behavior (33). Therefore, the combination of the two models not only helps to improve the limitations of existing theories on health behavior and helps to explain health behaviors to a greater degree, but also provides more recommendations for healthy behavior management. For myopia preventive behaviors, applying an integrative model to design a health education program should not only take into account parents' confidence in helping children adopt healthy behaviors, the behaviors and expectations of their friends and family, but also consider that parents' low awareness and beliefs may also influence their decisions. The combination of HBM and TPB has many applications in explaining parents' intentions to vaccinate their children and parental beliefs related to children's behavioral problems (20, 34). However, there is still a lack of exploration of the combination of two theories in explaining parents' intentions toward preschool children's myopia preventive behaviors. Therefore, based on the integrated model of HBM and TPB, this study explores the influencing factors of parents' intentions toward preschool children's myopia preventive behaviors, with the goal of exploring effective intervention measures to improve myopia preventive behavior and reduce the occurrence of myopia in preschool children in China.

## Materials and methods

### Study design and population

This cross-sectional study recruited parents of preschool children from seven preschools in Minhang District, Shanghai in January 2022. According to the calculation method of the sample size of the cross-sectional study, the average score of behavioral intention is 3.47, and the standard deviation is 0.94 (35). The significance level is 0.05 and allowable error was taken as 0.015 mean score, the sample size was required to be at least 1,253. With a possible invalid rate of 20%, no fewer than 1,566 participants should be recruited. There are 189 preschools in Minhang District with about 71,000 students. This study adopted the method of cluster sampling and recruitment invitations were sent to 189 preschools. Seven preschools were recruited on a first-come, first-served basis. Finally, a total of 1,628 parents of preschool children from seven preschools were included. The inclusion criteria for this study including (1) parents are guardians of children; (2) parents of children aged 3–6; (3) be able to complete the questionnaire independently. Exclusion criteria include (1) parents who have participated in other myopia intervention programs in the past 12 months; (2) parents of children with severe eye or head trauma. Parents of each preschooler were invited to complete an online questionnaire, the content of the questionnaire and the purpose of the study were explained to all parents prior to the start of the survey. Before participating in this study, all participants had only received the simplest school-based education on myopia knowledge required by the government, and had not received special training in myopia intervention. Informed consent was obtained from the parents.

In order to ensure the quality of the questionnaires filled in by parents, we set up two quality control questions. A questionnaire was considered valid only if all two quality control questions were answered correctly. In addition, the questionnaire was set to be submitted only after all questions have been answered, and each account could only submit the questionnaire once. In the end, a total of 1,537 questionnaires were collected, with a response rate of 94.41%, of which 1,300 were valid questionnaires, with a valid rate of 84.58%. The study was approved by the Medical Research Ethics Committee of the School of Public Health, Fudan University (The international registry nos. IRB00002408 and FWA00002399).

### Measurement

The questionnaires used in this study were developed by the research team after referencing to the questionnaire framework in the relevant literature. We mainly made some modifications to the behavioral content in the questionnaire to make it more in line with our research purposes, the question frames in different dimensions were retained, and none of the existing items were used. The 24 items of the HBM variables referred to two studies on parental intentions of children (20, 36). For example, perceived benefits and perceived barriers were measured using items like “It is good for my child to [target behavior],” “It is difficult for me keep my child [target behavior].” The content of the questionnaire for TPB variables was mainly based on a study about parents' decisions for limiting their young child's screen time (37). For example, items like “Most people who are important to me think that I should ensure that [target behavior]” were used to measure subjective norms. All items in the HBM and TPB models use a 5-point Likert scale and response scale from 1 “strongly disagree” to 5 “strongly agree.” Each dimension is expressed using the mean score, calculated by adding up the score for each item under each dimension and dividing by the corresponding number of items.

#### Socio-demographic characteristics

Data regarding child gender, child's age, preterm birth, single child, father's age, mother's age, father's educational level, mother's educational level, annual household income, father myopia and mother myopia were obtained from each participant.

#### HBM variables

The HBM variables include (Table 1): perceived susceptibility (2 items), perceived severity (6 items), perceived benefits (6 items), perceived barriers (5 items), and cues to action (5 items). For our study, Cronbach's alpha was 0.83, indicating acceptable internal consistency reliability.

**Table 1:** Exploratory factor analysis, descriptive analysis and reliability of the HBM and TPB variables.

**Study variables**	**Item**	**Mean**	**SD**	**Item loading**	^a^
Perceived susceptibility		3.38	0.77		0.625
	My child is at risk of developing myopia (or worsening myopia)	3.08	0.93	0.837	
	My child will be at increased risk for myopia (or myopia progression) without proper myopia prevention measures	3.69	0.87	0.800	
Perceived severity		3.87	0.90		0.914
	It is serious If my child develops myopia (or worsens myopia) that affects his daily activities	3.66	1.09	0.821	
	It is serious if my child develops myopia (or worsens myopia) that affects his studies	3.86	1.09	0.888	
	It is serious if my child develops myopia (or worsens myopia) that affects his appearance	3.49	1.18	0.797	
	It is serious if my child develops myopia (or worsens myopia) that affects his mental health	3.92	1.12	0.830	
	It is serious if my child develops myopia (or worsens myopia) that lead to eye disease such as retinal detachment in the future	4.37	0.96	0.645	
	It is serious if my child develops myopia (or worsens myopia)	3.90	1.03	0.845	
Perceived benefits		4.36	0.63		0.932
	It is good for my child to protect his/her vision by accumulating < 1 h of screen time every day	4.24	0.83	0.679	
	It is good for my child to take a break or look into the distance after watching a screen for a while to protect his/her vision	4.43	0.69	0.863	
	It is good for my child to protect his/her vision by accumulating < 1 h of reading and writing every day	4.21	0.79	0.724	
	It is good for my child to take a break after reading, writing, drawing or looking into the distance to protect his/her vision	4.41	0.67	0.901	
	It is good for my child to protect his/her vision by keeping good eye habits (such as not watching TV for too long, keeping enough distance, maintaining a good reading posture, etc.)	4.44	0.67	0.898	
	It is good for my child to protect his/her vision by reaching 1 h of outdoor activities every day	4.42	0.67	0.884	
Perceived barriers		1.70	0.75		0.861
	It is difficult for me to limit my child's screen time to no more than 1 h a day	1.90	1.05	0.757	
	It is difficult for me to get my kids to maintain good eye habits (such as not watching TV for too long, keeping enough distance, maintaining a good reading posture, etc.)	1.64	0.92	0.862	
	It is difficult for me to limit my child's reading, writing and drawing to no more than 1 h a day	1.65	0.90	0.794	
	It is difficult for me to keep my child's eye habit when reading and writing (for example, when writing homework, the distance between the book and the eyes should be 30–40 cm, do not lie down to read, etc.)	1.68	0.85	0.829	
	It is difficult for me to keep my kids outdoors for at least 1 h a day	1.63	0.92	0.718	
Cues to action		2.67	0.69		0.817
	Educational courses or lectures on myopia prevention in preschool children would motivate me to protect my child's vision	2.16	0.92	0.704	
	Paper brochures, books, newspapers and magazines related to the prevention and treatment of myopia in preschool children would motivate me to protect my child's vision	2.50	0.91	0.848	
	The popular science knowledge on the prevention and treatment of myopia in preschool children on the Internet would motivate me to protect my child's vision	2.72	0.84	0.835	
	Relatives, friends, colleagues, elders, etc. have told me about the prevention and treatment of myopia in preschool children, which would motivate me to protect my child's vision	2.56	0.93	0.795	
	The preschool has provided information on the prevention and treatment of myopia in preschool children (such as the issuance of relevant manuals on myopia prevention, or the teacher will regularly remind me to pay attention to the child's eye behavior at home, etc.), which would motivate me to protect my child's vision	3.41	0.92	0.560	
Attitudes		4.24	0.75		0.822
	My child should not accumulate more than 1 h of screen time per day	4.15	0.99	0.721	
	My child's cumulative reading, writing and drawing time should not exceed 1 h per day	3.94	1.01	0.720	
	My child should maintain good eye habits	4.46	0.84	0.737	
	My child should not spend < 1 h outdoors per day	4.39	0.88	0.716	
Subjective norms		4.03	0.86		0.946
	Most people who are important to me or my child think I should be concerned about my child's vision health	4.05	0.94	0.792	
	Most of the people who are important to me or my child think I should take steps to protect my child's vision	4.07	0.96	0.855	
	Most people who are important to me or my children think I should limit my child's cumulative screen time to no more than 1 h per day	4.03	0.95	0.867	
	Most people who are important to me or my children think I should limit my child's cumulative reading, writing, and drawing time to no more than 1 h per day	3.82	1.07	0.780	
	Most people who are important to me or my child think I should keep my child's good eye habits	4.14	0.91	0.826	
	Most people who are important to me or my children think I should keep my children outdoors for at least 1 h a day	4.07	0.95	0.805	
Perceived behavioral control		3.50	1.04		0.903
	For the next year, I am confident to limit my child's cumulative screen time to no more than 1 h per day	3.41	1.21	0.805	
	For the next year, I am confident to limit my child's cumulative reading, writing, and drawing time to no more than 1 h per day	3.37	1.22	0.808	
	For the next year, I am confident to ensure that my child develops good eye habits (such as not watching TV for too long, keeping enough distance, maintaining a good reading posture, etc.)	3.58	1.12	0.842	
	For the next year, I am confident that my kids will be outdoors for at least 1 h a day	3.62	1.16	0.757	

a Cronbach's alpha; HBM a = 0.83; TPB a = 0.92; integrative model a = 0.91.

SD, standard deviation.

#### TPB variables

The TPB variables include (Table 1): attitude (4 items), subjective norms (6 items), perceived behavioral control (4 items). For our study, Cronbach's alpha was 0.92, implying good internal consistency reliability.

#### Intention

Parents' intentions toward preschool children's myopia preventive behavior were measured by 4 items, including: “I intend to take measures to protect my child's vision in the next 12 months,” “I intend to limit my child's screen time to no more than 1 a day in the next 12 months,” “I intend to limit the time my child spends reading, writing, and drawing to no more than 1 h a day in the next 12 months,” “I intend to keep my child's outdoor time at least 1 h a day in the next 12 months.” Questions were scored on a 5-point rating scale ranging from 1 (strongly disagree) to 5 (strongly agree). Higher scores reflected higher levels of parents' intentions.

### Statistical analysis

Numbers and percentages were used to describe qualitative variables, while quantitative variables were presented as mean and standard deviation. Cronbach's alpha was calculated to test the internal consistency of each dimension in the HBM and TPB models. Use *t*-test or ANOVA to analyze differences in parental intention, where appropriate. Exploratory factor analysis was used to examine the construct validity and factors were extracted by applying principal component analysis with a varimax rotation. Items with factor loadings <0.40 or loadings >0.40 on multiple factors were eliminated (38). To explore the relationship between independent variables and parents' intentions toward preschool children's myopia preventive behaviors, a hierarchical multiple regression was performed. Model 1 consisted of significant socio-demographic variables found in the univariate analysis. Model 2 entered perceived susceptibility, perceived severity, perceived benefits, perceived barriers, and cues to action; these were HBM variables. Model 3 entered TPB variables, which were attitude, subjective norms and perceived behavioral control. The order of variables inclusion in the model was based on two prior hypotheses. First, HBM variables can explain additional variance after accounting for individual socio-demographic factors. Second, after accounting for the variance of individual factors and HBM variables, the increased variance may be explained by the TPB variables. All statistical analysis were performed using IBM SPSS Statistics 20 software (IBM Corp.). Statistical significance was set at 0.05.

## Results

### Exploratory factor analysis, descriptive analysis and reliability of the HBM and TPB variables

The Bartlett test of sphericity (χ^2^ = 36208.14, *p* < 0.001) and Kaiser–Meyer–Olkin measure (0.91) verified interpretability of the exploratory factor analysis. Eight factors with eigenvalues >1 were retained, explaining 69.24% of the total variance. Two questionnaire items were eliminated: “It is serious If my child develops myopia (or worsens myopia) that increases expenses” (perceived severity) and “The ophthalmologist once told me to protect my child's vision” (cues to action). Besides, the factor loadings for the remaining items ranged from 0.560 to 0.901, which is acceptable (Table 1). The Cronbach's alpha coefficient of perceived susceptibility, perceived severity, perceived benefits, perceived barriers, cues to action, attitude, subjective norms and perceived behavioral control were 0.625, 0.914, 0.932, 0.861, 0.817, 0.822, 0.946, and 0.903, respectively, indicating good internal consistency reliability. The mean scores and standard deviation for the HBM and TPB variables are detailed in Table 1.

### Differences among sample socio-demographic and parental intention

A total of 1,300 parents of preschool children aged 3–6 years were recruited for this study. Table 2 presents the socio-demographic characteristics of the children and parents. The average age of the fathers was 35.79 (SD = 4.40) years, and the average age of the mothers was 34.16 (SD = 4.01) years. About half are single-child families (53.0%). Most of the parents have a bachelor's degree or above. The self-reported prevalence of myopia was 49.9% for fathers and 54.5% for mothers. Among these socio-demographic variables, univariate analysis found there were significant differences in parental intention scores in child's age (*F* = 2.75, *p* = 0.042).

**Table 2:** Relationships between the parents' intentions scores and socio-demographic characteristics for children and parents (*n* = 1,300).

**Characteristics**	***N* (%)**	**Parental intention score,** **mean ±SD**	***t*/*F***	***P***
Child's gender			0.38	0.702
Male	676 (52.0%)	3.92 ± 0.82		
Female	624 (48.0%)	3.90 ± 0.81		
Child's age			2.75	0.042
3	226 (17.4%)	4.00 ± 0.75		
4	409 (31.5%)	3.94 ± 0.81		
5	497 (38.2%)	3.90 ± 0.84		
6	168 (12.9%)	3.77 ± 0.84		
Preterm birth (< 37 weeks)			1.06	0.288
Yes	112 (8.6%)	3.92 ± 0.82		
No	1,188 (91.4%)	3.80 ± 0.82		
Single child			1.52	0.130
Yes	689 (53.0%)	3.95 ± 0.80		
No	611 (47.0%)	3.88 ± 0.83		
Father's age			1.16	0.315
24–30 years	135 (10.4%)	4.01 ± 0.83		
31–40 years	973 (74.8%)	3.91 ± 0.82		
41–49 years	192 (14.8%)	3.88 ± 0.80		
Mother's age			2.27	0.103
20–30 years	221 (17.0%)	4.02 ± 0.79		
31–40 years	1,000 (76.9%)	3.89 ± 0.82		
41–47 years	79 (6.1%)	3.87 ± 0.80		
Annual household income (CNY¥)			1.55	0.213
≤ 100,000	239 (18.4%)	3.86 ± 0.85		
100,001–300,000	552 (42.4%)	3.89 ± 0.80		
≥300,001	509 (39.2%)	3.96 ± 0.82		
Father's educational level			1.01	0.363
High school or below	268 (20.6%)	3.97 ± 0.82		
College or university	957 (73.6%)	3.91 ± 0.82		
Postgraduate	75 (5.8%)	3.83 ± 0.80		
Mother's educational level			0.45	0.636
High school or below	263 (20.2%)	3.94 ± 0.84		
College or university	965 (74.3%)	3.91 ± 0.82		
Postgraduate	72 (5.5%)	3.84 ± 0.74		
Father myopia			−0.15	0.881
Yes	649 (49.9%)	3.91 ± 0.81		
No	651 (50.1%)	3.92 ± 0.82		
Mother myopia			−0.62	0.535
Yes	708 (54.5%)	3.90 ± 0.80		
No	592 (45.5%)	3.93 ± 0.84		

Income is presented in Chinese yuan.

N, number; SD, standard deviation.

### Hierarchical multiple regression

A hierarchical multiple regression analysis was conducted to investigate the association between child's age, HBM variables, TPB variables and the parents' intentions toward preschool children's myopia preventive behaviors (Table 3). Multicollinearity analysis was performed on all independent variables, and all variance inflation factor were <1.34. In model 1, Child's age was entered as a control variable and explained 0.6% of the variance (*F* = 7.241, *p* = 0.007). When the HBM variables were entered in model 2, the proportion of variance increased to 25.4% (*F* = 73.290, *P* < 0.001). In model 3, TPB variables were entered and explained 63.2% of the variance (*F* = 246.076, *p* < 0.001). It was found that perceived severity (β = 0.049, *p* = 0.008), perceived barrier (β = −0.083, *p* < 0.001), attitude (β = 0.145, *p* < 0.001), subjective norms (β = 0.230, *p* < 0.001) and perceived behavioral control (β = 0.507, *p* < 0.001) were significantly associated with parents' intentions toward preschool children's myopia preventive behaviors.

**Table 3:** Hierarchical multiple regression analysis including age, HBM variables and TPB variables.

**Variables**	**Model 1**		**Model 2**		**Model 3**	
	**B (SE)**	**Beta (β)**	**B (SE)**	**Beta (β)**	**B (SE)**	**Beta (β)**
Intercept	4.207 (0.111)**		1.906 (0.195)**		0.616 (0.144)**	
Age	−0.066 (0.024)	−0.074**	−0.042 (0.021)	−0.047	0.003 (0.015)	0.003
Perceived susceptibility			0.032 (0.026)	0.030	0.033 (0.018)	0.031
Perceived severity			0.130 (0.024)	0.144***	0.045 (0.017)	0.049**
Perceived benefits			0.353 (0.035)	0.270***	0.036 (0.027)	0.028
Perceived barriers			−0.292 (0.027)	−0.267***	−0.090 (0.020)	−0.083***
cues to action			0.203 (0.029)	0.171***	0.019 (0.021)	0.016
Attitudes					0.158 (0.023)	0.145***
Subjective norms					0.219 (0.021)	0.230***
Perceived behavioral control					0.399 (0.017)	0.507***
*R* ^2^	0.006**		0.254***		0.632***	
*F*	7.241**		73.290***		246.076***	
Δ*R*^2^	0.006**		0.248***		0.378***	
ΔF	7.241**		86.025***		441.752***	

^**^P < 0.01, ^***^P < 0.001; B, unstandardized regression coefficient; SE, standard error of unstandardized regression coefficient; β, standardized regression coefficient.

## Discussion

This study comprehensively used HBM and TPB theories to explore the influencing factors of parents' intentions toward preschool children's myopia preventive behaviors. The final integrative model showed that perceived severity, perceived barriers, attitudes, subjective norms, and perceived behavioral control were associated with parents' intentions. The present study found that the combination of the two theories significantly improved the degree of explanation of parents' intentions toward preschool children's myopia preventive behaviors.

Child's age was the only significant sociodemographic variable in the univariate analysis. Increasing children's age was associated with a decline in parents' intentions toward preschool children's myopia preventive behaviors. An intervention study of preschool children have found that parents report increasing difficulty controlling their children's time spent outdoors and screen use as children get older (39). This decline in parental control as children age might contribute to the decrease in parents' intentions, suggesting that parents should supervise their children to form good myopia preventive behaviors in their early years.

Consistent with other research findings, there was a significant positive association between perceived severity and parents' intentions toward preschool children's myopia preventive behaviors (17). As such, the higher level of perceived severity of myopia, the greater intention parents would exhibit to help their children take myopia preventive behaviors. A previous study further supported this result, where parents with higher levels of severity perception were more likely to supervise children to reduce screen time (40). Besides, this study found perceived severity to be a less influential factor, possibly because the public tends to view myopia as a non-life-threatening condition, distinct from infectious and degenerative diseases such as measles and cancer (17). However, the importance of perceived severity cannot be overlooked, since the intention to act against the threat is not activated if parents perceive myopia as not a threat (26, 41).

Perceived barrier is another variable significantly correlated with parents' intentions toward preschool children's myopia preventive behaviors in HBM theory, which is consistent with other research findings (17). When parents realize there are barriers to helping their children maintain good myopia preventive behaviors, their intentions decrease. Importantly, time cost has been reported as a major barrier in other studies (39). Most families are dual-employed parents who are too busy with their jobs to supervise their children's screen viewing or spend time with their children in outdoor activities (39). However, the average level of parents' perceived barriers was not high in this study. This may indicate that for most preschool children, it is not difficult for parents to control them to adopt various types of myopia preventive behaviors. Nonetheless, perceived barrier shows a significant effect in the final integrative model, possibly due to the strong correlation between perceived barrier and health protective behaviors (42). Although the effect size of perceived barrier on parents' intentions may be weaker than other variables in the integrative model, it still suggests that intervention on parents' perceived barrier can play a role. Therefore, we still recommend that appropriate educational training should be conducted to improve parental intervention techniques and strategies for children, while advocating for parents to spend more time helping children adopt myopia preventive behaviors.

Our study demonstrated a significant positive relationship between parental intentions and attitudes toward preschool children's myopia preventive behaviors. Given the crucial influence of parents on children's lifestyles, parental attitudes are considered to be a key agent in controlling the development of children's myopia (43). Therefore, improving parents' awareness of myopia and changing their attitudes will help motivate parents to take active measures to prevent children's myopia.

The relationship between subjective norms and intentions was often considered to be weak (44). However, our findings suggested that subjective norms not only have a significant positive correlation with parents' intentions toward preschool children's myopia preventive behaviors, but also explain intentions in a large degree. This is consistent with a study finding that subjective norm is a strong predictor of parents' intentions to limit their child's screen time behavior (37). This may be explained by the fact that parents are perceived to have parenting responsibilities and are responsible for their children's healthy behaviors, thereby reinforcing parents' perceived social norms (45, 46). In practical terms, this study provides evidence that subjective norms play an important role in involving parents' intentions toward preschool children's myopia preventive behaviors. Based on the apparent influence of subjective norms, it can be hypothesized that directing social change in a positive direction will be effective, especially emphasizing the advocacy of myopia preventive behaviors by influential people (e.g., teachers). This suggested that it is necessary to provide teachers and parents with appropriate training in children's vision health. Similarly, emphasizing to parents that more and more people are paying attention to maintaining myopia preventive behaviors in their children can have a positive effect.

In this study, the perceived behavioral control component showed the strongest effect on parents' intentions toward preschool children's myopia preventive behaviors. This was supported by a previous study revealing that perceived behavioral control was the main predictor of behavioral intentions (31). The more resources individuals have and the fewer obstacles they anticipate, the greater control they perceived over their behaviors, and the stronger confidence they have to perform behaviors (22). Therefore, when parents have a more comprehensive understanding of children's vision health and myopia preventive behaviors, their confidence in controlling children's related behaviors will increase, and they will be more willing to take action. Accordingly, parents' perceived behavioral control should be strengthened through enhanced advocacy and practice.

Based on the survey results, most parents are aware of their children's possible risk of myopia and the benefits of myopia preventive behaviors, and feel a general level of health cues. But this did not increase their intentions to adopt myopia preventive behaviors for their children. Previous studies based either on TPB or HBM to explain parents' intentions to vaccinate their children found that not all TPB and HBM constructs had significant effects, with some variables showing significant effects in one study and others in another study showing a significant impact (20, 25, 47–49). Just as the findings of this study that perceived susceptibility, perceived benefits and cues to action were not significant variables are inconsistent with other findings on parents' intentions toward preschool children's myopia preventive behaviors (17, 30, 40). It is not surprising that these results are different from those of other populations. Given that few studies have explored factors related to parents' intentions toward preschool children's myopia preventive behaviors using TPB and HBM constructs simultaneously, interventions should be designed based on both models to further verify the effects of each construct. Overall, the integrated model of HBM and TPB can better explain the effects of variables on parents' intentions toward preschool children's myopia preventive behaviors compared to using each of the two behavior models separately.

There are several limitations for this study to consider. First, the participants were recruited from Minhang District, Shanghai, which is a city with a high level of economic and urbanization. This might limit the generalization of the findings to populations in other regions, particularly rural areas. Second, the data of this study were collected through an online survey, which means that there may be some situations that affect the quality of the data, such as comprehension bias in the content of the questionnaire and fill out the questionnaire without reading it. However, we avoid these problems by answering questions from participants in real time online, and setting quality control questions. In addition, to ensure the smooth conduct of research, the use of online surveys during a pandemic is reasonable. Third, this study did not collect data on the refractive errors/ prevalence of myopia of the children recruited as this may have influenced parental knowledge of myopia. Future research may consider incorporating these factors. Finally, although the integrated model of HBM and TPB used in this study can explain parents' intentions toward preschool children's myopia preventive behaviors well. However, other theoretical models of behavior change such as Self-Determination Theory and Integrated Behavioral Models were not included in the study. Future research can incorporate more theoretical models to explore.

## Conclusions

The integrated model of HBM and TPB constructed in this study could be well used to explain parents' intentions toward preschool children's myopia preventive behaviors. Parents' perceived severity, perceived barriers, attitudes, subjective norms, and perceived behavioral control can be prioritized intervention targets for myopia preventive practices in preschool children. In particular, there should be more advocacy and practical training for the public and parents of preschool children. Not only to promote myopia preventive behaviors and expectations of the public, but also to improve parents' awareness and attitude, thereby increasing the parents' intentions.

## Data availability statement

The raw data supporting the conclusions of this article will be made available by the authors, without undue reservation.

## Ethics statement

The studies involving human participants were reviewed and approved by the Medical Research Ethics Committee of the School of Public Health, Fudan University (The international registry nos. IRB00002408 and FWA00002399). Written informed consent to participate in this study was provided by the participants' legal guardian/next of kin.

## Author contributions

NJ, JC, and HC were responsible for the acquisition, analysis and interpretation of data, and the drafting of the manuscript. YL and YZ contributed to the acquisition, interpretation of data, and critically reviewed the manuscript for important intellectual content. QW, TW, HZ, and HL provided advice regarding study design and developed the questionnaire. JW and JC were the project coordinator and contributed to the review and revision of the manuscript. All authors read and approved the final manuscript.

## Funding

This study was supported by the Minhang District Natural Science Research Project of Shanghai (Grant No. 2021MHZ047), the Shanghai Eye Starlight Children and Adolescent Myopia Prevention and Control Personnel Training Program (Grant No. 2022HYXG-SQ01), and the Fudan-Minhang Health Consortium Cooperation Project (Grant No. 2021FM10).

## Conflict of interest

The authors declare that the research was conducted in the absence of any commercial or financial relationships that could be construed as a potential conflict of interest.

## Publisher's note

All claims expressed in this article are solely those of the authors and do not necessarily represent those of their affiliated organizations, or those of the publisher, the editors and the reviewers. Any product that may be evaluated in this article, or claim that may be made by its manufacturer, is not guaranteed or endorsed by the publisher.
